# Is bouldering-psychotherapy a cost-effective way to treat depression when compared to group cognitive behavioral therapy – results from a randomized controlled trial

**DOI:** 10.1186/s12913-021-07153-1

**Published:** 2021-10-26

**Authors:** Larissa Schwarzkopf, Lisa Dorscht, Ludwig Kraus, Katharina Luttenberger

**Affiliations:** 1 IFT Institut für Therapieforschung, Leopoldstrasse 175, 80804 Munich, Germany; 2 Institute of Health Economics and Health Care Management, Helmholtz Zentrum München GmbH, German Research Center for Environmental Health, Ingolstaedter Landstrasse 1, 85764 Neuherberg, Germany; 3 Center for Health Services Research in Medicine, Department of Psychiatry and Psychotherapy, University Hospital Erlangen, Friedrich-Alexander University Erlangen-Nürnberg (FAU), Schwabachanlage 6, 91054 Erlangen, Germany; 4 Department of Public Health Sciences, Centre for Social Research on Alcohol and Drugs, Stockholm University, 10691 Stockholm, Sweden; 5 Institute of Psychology, ELTE Eötvös Loránd University, Kazinczy utca 23-27, 1075 Budapest, Hungary

**Keywords:** Affective disorder, Psychotherapeutic treatment, Therapeutic climbing, Health economic evaluation, Comparative effectiveness, Health care expenditures, Productivity loss

## Abstract

**Background:**

Bouldering-Psychotherapy (BPT) has proven to effectively reduce depressive symptoms, but evidence on its cost-effectiveness is lacking. Corresponding information is paramount to support health policy decision making on a potential implementation of BPT in routine care.

**Methods:**

Using data from the German KuS trial BPT was compared with group Cognitive Behavioral Therapy (CBT). Severity of depression symptoms at end of the intervention was operationalized via Montgomery-Asberg Depression Rating Scale (MADRS) and Patient Health Questionnaire (PHQ-9). Adopting a societal perspective, direct medical costs and productivity loss were calculated based on standardized unit costs. To determine incremental cost-effectiveness ratios (ICER) and cost-effectiveness-acceptance curves (CEAC), adjusted mean differences (AMD) in costs (gamma-distributed model) and both effect parameters (Gaussian-distributed model) were obtained from 1000 simultaneous bootstrap replications.

**Results:**

BPT was related to improved effects (AMDs: MADRS -2.58; PHQ-9: − 1.35) at higher costs (AMD: +€ 754). No AMD was significant. ICERs amounted to €288 per MADRS-point and €550 per PHQ-9-point. For both effect parameters about 20% of bootstrap replications indicated dominance of BPT, and about 75% larger effects at higher costs. At hypothetical willingness to pay (WTP) thresholds of €241 (MADRS) and €615 (PHQ-9) per unit of change BPT had a 50% probability of being cost-effective.

**Conclusion:**

BPT is a promising alternate treatment strategy which – in absence of established WTP thresholds for improving symptoms of depression – cannot unambiguously be claimed cost-effective. Further studies defining subgroups that particularly benefit from BPT appear paramount to delineate recommendations for an efficient prospective roll-out to routine care.

**Supplementary Information:**

The online version contains supplementary material available at 10.1186/s12913-021-07153-1.

## Background

Over the last decade, prevalence of depression has considerably increased not only in Germany [1, 2] but also worldwide [3]. In 2017, the point prevalence of depression in Germany’s Statutory health insured (SHI) population aged ≥15 years, was estimated at 15.7%, translating into about 11.3 million individuals diagnosed in the resident population [1]. Globally, the number of individuals concerned was estimated at 258 million [3]. Being furthermore listed among the top 5 causes of years lived with disability (2016: 44.2 million YLDs) [4] depression represents a major public health concern.

Besides morbidity aspects depression is associated with substantial economic burden, mainly in context of productivity losses. In 2014, a German study estimated depression-associated mean annual per capita costs at €3813, of which 27.9% were indirect costs. Moreover, this analysis unveiled, that costs for severe depression (€6,302) are more than twice the costs of moderate (€2971) or mild depression (€3036). Based on the Germany-specific prevalence of the distinct severity levels, annual economic burden of depression was estimated at €15.6 billion [5]. A recent meta-analysis supports this piece of evidence by indicating that depression is related to significant direct excess costs across all age groups and to significant indirect excess costs in non-elderly adults [6].

These findings stress the relevance of comprehensive, early depression treatment to avoid disease progression or even chronification. Here, besides pharmacological treatment, different psycho-therapeutic approaches covering especially cognitive behavioral therapy (CBT) are considered state of the art [2, 7, 8]. Since the antidepressant effect of physical exercise has been shown to be comparable to psychotherapy and antidepressant psychotropic drugs [9–11], current guidelines additionally recommend physical activity [2, 8]. As bouldering/climbing has proven its positive impact on psychological wellbeing [12, 13], concepts of “therapeutic climbing” are gaining interest in depression treatment. Recent systematic reviews suggest that it might be the most important step to get patients involved into a therapeutic program as differences between the different approaches are small [14]. With therapeutic climbing as less stigmatizing therapy option a new group of patients could be reached.

Despite early studies emphasize the beneficial impact of corresponding approaches [15–19], high quality randomized controlled trials (RCTs) that substantiate these findings are widely lacking. Combining bouldering with psychotherapeutic elements (so-called Bouldering Psychotherapy/BPT) [20] is a subsequent step to harness synergies of both concepts. Previous RCTs with active and passive controls already demonstrated that BPT achieves a reduction of depression-related symptom burden [21, 22]. However, its comparative effectiveness in relation to CBT has not been systematically examined before.

Considering furthermore limited resources to fund, comparative effectiveness per se cannot be the sole decision criterion to comprehensively judge the added value of any intervention. Even more, additional costs and additional effects ought to form an acceptable ratio, referred to as cost-effectiveness. Based on data from the German multi-center KuS RCT (Klettern und Stimmung, i.e. “Climbing and Mood”) [23] this paper elucidates cost-effectiveness of a ten weeks BPT-program using group CBT (gold-standard) as the comparator.

## Methods

### Study design and participant recruitment

Details on the KuS RCT can be obtained from the study protocol [23]. Briefly, KuS was designed as three-armed randomized (stratified by sex and depression severity), controlled, single blind multi-center trial, with patient recruitment in the metropolitan area of Nuremberg/Erlangen/Fuerth, the rural Weyarn area and the capital of Berlin. At Nuremberg/Erlangen/Fuerth and Weyarn the intervention groups (BPT or CBT) and the active control group (home-based exercise program/ EP) were conducted within four consecutive waves, at Berlin two waves took place. The study was performed in accordance with the declaration of Helsinki and approved by the Ethics Committee of Friedrich-Alexander-Universität Erlangen-Nürnberg (Ref. 360_16 B) and registered in parts retrospectively with the trial identification number ISRCTN12457760 in June 2017.

Recruitment took place between March 2017 and March 2018. Study participants had to fulfill following inclusion criteria: age ≥ 18 years, depressive symptoms, Body Mass Index (BMI) between 17.5 and 40, and ability to reach any therapy location as well as no parallel enrolment in another psychotherapeutic group therapy, no initiation of treatment with psychotropic drugs or individual psychotherapy within the last 2 months, no planned inpatient stay during the intervention period, no physical contraindications for bouldering, absence of distinct psychiatric disorders, and no acute suicidality. After provision of written informed consent, interested individuals fulfilling these criteria were randomly assigned to either one of the intervention groups or the active control group. For all groups, intervention took place between May 2017 and June 2018.

Comparison of BPT and EP relied on the hypothesis of superiority (for results see [21]), comparison of BPT and CBT on that of non-inferiority. As several recommendations for health economic evaluations suggest choosing the current standard of care as comparator for cost-effectiveness analyses [24–26], this paper contrasts BPT (“innovation”) with CBT (“gold-standard”) and disregards EP.

### Interventions

Both, BPT and CBT comprised ten consecutive two-hours sessions delivered over a 10-weeks period in groups of up to eleven participants. Detailed manuals with fixed schedules for the distinct sessions were developed for both interventions. Each session addressed a specific topic considered relevant for mitigating symptoms of depression. Regarding CBT, the manual builds on established treatment plans [27–29] including mindfulness and body relaxation exercises. The BPT manual followed a standardized procedure and addressed mindfulness exercises, psychoeducational elements, topic-related bouldering exercises under therapeutic supervision, exchange of individual experiences between participants and transfer to daily life, body-related relaxation exercises, and free bouldering. A detailed description of both interventions is described elsewhere [23]. After each session, therapists filled protocol assessment surveys to document treatment adherences.

### Effects

Effects portrayed in the cost-effectiveness analyses mirror the primary trial outcome “severity of depression symptoms” which was once assessed with the Montgomery-Asberg Depression Rating Scale (MADRS) and once with the Patient Health Questionnaire (PHQ-9).

Our primary analysis targeted at MADRS. This scale assesses severity of depression based on ten items, each rated on a six-level Likert-Scale [30]. Total scores of ≥31 points indicate severe depression and scores < 10 are considered as remission [31, 32]. The established minimal important clinical difference (MID) is a change of ≥3 points [33, 34]. Our secondary analysis addressed PHQ-9. This tool assesses severity of depression via 9 items, each measured on a three-level Likert-Scale, with total scores of ≥20 points indicating severe depression and scores < 8 points reflecting remission [35, 36]. Here the established MID is a change of ≥1.9 points [37].

### Costs

Depression-related resource utilization was assessed by a modified version of the validated FIMA questionnaire [38, 39] targeting at disease-specific instead of all-cause health care utilization. At baseline (t0) the questionnaire refers to the 3 months prior to start of the intervention. At end of the intervention (t1), it reflects the ten-weeks intervention period.

To assess direct medical cost, we considered outpatient physician care (general practitioners and psychiatrists), inpatient psychiatric care (inpatient stay in a psychiatric ward, visits of psychiatric day clinics, depression-related rehabilitation), psychotherapy (single and group sessions) and anti-depressants taken. Indirect costs were operationalized as a combination of days of work absenteeism (depression-related sick-leave), reduced working hours (depression-related part-time work) and early retirement owing to depression. Adopting a societal perspective, costs were calculated from the patients’ self-reported resource utilization, multiplied with standardized resource-specific unit costs [40], which were up-dated via source-research to the base year 2017. Indirect costs reflected a human capital approach and relied on official German statistics on average annual as well as hourly compensation of employees and potential working days in 2017 [see Table S1].

As these unit costs do not reflect psychotherapists’ treatment in sufficient detail, we applied the 2017 schedule of fees for psychotherapists’ services [41] to price corresponding resource utilization. For pricing of anti-depressants, we identified the distinct drugs based on substance-names and applied the pharmacy retail price of the largest package by the cheapest provider obtained from the chargeable masterfile of the SHI drug price index which is available from the Scientific institute of the AOK SHI Funds.

Intervention costs per patient were calculated based on the study documentation and account for group therapy (CBT and BPT) as well as for entrance fees for the bouldering gym, rental fees for climbing shoes if required, and a one-time safety training for therapists which was evenly distributed across the study participants (all BPT only).

### Statistical analyses

The main intention to treat (ITT) analysis included those 156 randomized patients who entered the study. We observed completely missing t1 data in 7 BPT and 9 CBT members but no single missing items. Neither patient characteristics nor baseline MADRS, PHQ-9, and total costs were significantly associated with missingness. Hence, we assumed missingness at random. Means stemming from a multiple imputation with 10 data sets were applied to impute missing MADRS, PHQ-9 and cost data [42]. For a first descriptive analysis we compared unadjusted imputed means (along with standard deviations (SD)) for MADRS, PHQ-9, and total costs at t0. Corresponding comparisons were performed for unadjusted, unimputed quotas of service users in the distinct health care domains and the related unadjusted, unimputed mean per capita utilization. Categorical data were contrasted via Chi^2^-tests and continuous data via Wilcoxon-Mann-Whitney-tests.

To subsequently calculate adjusted effects at t1, we ran Gaussian-distributed generalized linear models (GLM) that considered baseline values of MADRS and PHQ-9, respectively, the randomization parameters sex and depression severity as well as study site (Berlin, Weyarn, Nuremberg/Erlangen/Fuerth), and wave as covariates. As study participants are clustered by wave and study site, statistical approaches that reflect this nested structure would have been preferable. Owing to issues of non-convergence, covariate adjustment was however the only means to address potential study site and wave effects. Then, BPT and CBT were contrasted using the model-based adjusted mean differences (AMD) of MADRS and PHQ-9 including two-sided 95% confidence intervals (CI).

Difference in total costs at t1 was investigated using a one-part gamma-distributed GLM with log-link to handle the right-skewed distribution of cost data [43, 44]. AMDs at t1 adjusted for baseline total costs, sex, depression severity, study site and wave were estimated using the method of recycled predictions with group (BPT/CBT) as the coefficient of interest [45]. Then, 95%-CIs were determined via 1000 non-parametric bootstrap replications [46].

Incremental cost-effectiveness ratios (ICER), defined as the AMD of total costs divided by the AMD of each effect parameter, and uncertainty around were estimated by 1000 simultaneous bootstrap replications of adjusted total costs and adjusted effects. These results were visualized in form of cost-effectiveness planes [46] and cost-effectiveness acceptance curves (CEAC). In absence of an established willingness-to-pay (WTP) threshold for MADRS and PHQ-9 change we assessed at which hypothetical threshold BPT would have a 50% probability of being cost-effective.

To judge the robustness of our results, we ran a sensitivity analysis (SA_1_) with those 129 individuals completing the interventions as per protocol (PP). In a second sensitivity analysis (SA_2_) we excluded the at t1 most expensive 1% of participants. This outlier-eliminated sample consisted of all 77 CBT and 77 BPT members.

All statistical analyses were performed with a significance level of 5% using SAS (SAS Institute Inc., Cary, NC, USA, version 9.4). Graphics were edited in RStudio (version 3.5.1).

## Results

### Study participants

Randomization achieved a well-balanced sample [see Table 1], which mainly comprised of female patients (67.3%) and individuals with moderate (38.4%) or moderate to severe (26.9%) depression. About half of the participants stemmed from the Nuremberg/Erlangen/Fuerth (48,7%) and roughly one third (30.1%) from the Berlin region.

**Table 1:** Baseline characteristics of the study sample

Variable	BPT (***n*** = 79)		CBT (***n*** = 77)		Total Sample (***n*** = 156)		***p***-value
Age, M (SD)	41.8	(12.6)	40.3	(11.4)	41.0	(12.01)	0.43
Sex, n (%) female	54	(68.4)	51	(66.2)	105	(67.3)	0.78
BMI, M (SD)	23.9	(3.37)	24.6	(4.17)	24.24	(3.79)	0.29
Family status, n (%)							
Single	40	(50.6)	43	(55.8)	83	(53.2)	0.46
Married/living in a partnership	23	(29.1)	24	(31.2)	47	(30.1)	
Separated/divorced/widowed	16	(20.3)	10	(13.0)	26	(16.7)	
School education, n (%)							
< 9 years	0	(0.0)	0	(0.0)	0	(0.0)	0.99
9 years	9	(11.4)	9	(11.7)	18	(11.5)	
10 years	18	(22.8)	17	(22.1)	35	(22.4)	
≥ 11 years	52	(65.8)	51	(66.2)	103	(66.0)	
Current occupation: yes, n (%)	50	(63.3)	45	(58.4)	95	(60.9)	0.54
Completed therapy in the past: yes, n (%)	59	(74.7)	52	(67.5)	111	(71.1)	0.35
PHQ, severity grade n (%)							
Mild (up to 9 points)	18	(22.8)	12	(15.6)	30	(19.2)	0.38
Moderate (10–14 points)	28	(35.4)	33	(42.9)	61	(38.4)	
moderate to severe (15 to 19 points)	19	(24.1)	23	(29.9)	42	(26.9)	
Severe (20 points and above)	14	(17.7)	9	(11.7)	23	(14.7)	
First depressive episode: yes, n (%)	19	(24.1)	19	(24.7)	38	(24.4)	0.93
Number of depressive episodes, n (%)							
1–2	23	(29.2)	19	(24.7)	42	(27.0)	0.97
3–4	18	(22.8)	22	(28.6)	40	(25.7)	
5–10	8	(10.2)	10	(13.0)	18	(11.4)	
> 10 or chronic depression (> 2 years)	4	(5.2)	2	(2.6)	6	(3.8)	
Study site, n (%)							
Erlangen/Nuremberg region	39	(49.4)	37	(48.1)	76	(48.7)	0.28
Weyarn region	20	(25.3)	27	(35.1)	47	(30.1)	
Berlin region	20	(25.3)	13	(16.9)	33	(21.2)	
MADRS, M (SD)	23.5	(8.93)	24.0	(7.69)	23.7	(8.32)	0.66
PHQ-9	13.7	(5.49)	13.8	(4.65)	13.7	(5.07)	0.86
Duration of intervention, M (SD)	11.4	(1.05)	11.6	(1.36)	11.5	(1.22)	0.23

*P*-value stemming from Chi^2^-tests for categorical and from t-test for continuous variables

*M* Mean, *MADRS* Montgomery-Asberg Depression Rating Scale, *SD* Standard deviation, PHQ-9 = Patient Health Questionnaire

During the on average 11.5 weeks study period we observed 11 dropouts among the 79 BPT members (13.9%) and 6 dropouts among the 77 CBT members (7.8%) [see Fig. S1]. Furthermore, 4 BPT and 6 CBT members presented protocol violations. These individuals were excluded for SA_1_ resulting in 64 BPT and 65 CBT members. PP sample and ITT sample did not substantially differ.

### Effects

At t0 mean unadjusted MADRS scores were comparable for BPT (23.5; SD = 8.93) and CBT (24.0; SD = 7.69) and so were mean unadjusted PHQ-9 scores (BPT: 13.7; SD = 5.49 | CBT: 13.8, SD = 4.65) [see Table 1].

Adjusted MADRS scores at t1 amounted to 15.8 [95%-CI: 13.5; 18.1] for BPT and to 18.4 [95%-CI 16.1; 20.8] for CBT. This translates to a not significant AMD of − 2.58 [95%-CI: − 5.52; 0.25], in favor of BPT. Regarding PHQ-9, at t1 an adjusted score of 9.4 [95%-CI: 8.0; 10.7] was observed in BPT and of 10.7 [95%-CI: 9.3; 12.1] in CBT. The resulting AMD of − 1.35 [95%-CI: − 2.97; 0.40] for the benefit of BPT was not significant [see Table 2].

**Table 2:** Unadjusted and adjusted outcome parameters at the end of the intervention period

	Mean unadjusted, imputed values with SD		Mean adjusted, imputed values with 95%-CI		
	BPT (***n*** = 79)	CBT (***n*** = 77)	BPT (***n*** = 79)	CBT (***n*** = 77)	AMD
**Effect parameter**					
MADRS	15.4 (9.1)	18.1 (10.4)	15.8 [13.5; 18.1]	18.4 [16.1; 20.8]	-2.58 [−5.52; 0.25]
PHQ-9	9.0 (5.5)	10.4 (6.0)	9.4 [8.0; 10.7]	10.7 [9.3; 12.1]	-1.35 [−2.97; 0.40]
**Cost parameter in €**					
Total costs	4624 (4793)	4199 (4395)	6019 [4255; 8629]	5266 [3684; 7972]	754 [− 1280; 2737]
Indirect costs	3726 (4705)	3418 (4396)	3410 [2493;4386]	4154 [3034; 5340]	− 744; [− 2247;660]
Direct costs	640 (563)	548 (591)	769 [581;1017]	510 [381;544]	259 [−3;544]
Intervention cost	257 (95)	233 (86)			

Adjustment for baseline value, sex, depression severity, study site and wave

Effect estimates based on Generalized Linear Model with Gaussian-distribution,

Cost estimates for total and direct costs based on one-part gamma-distributed model, cost estimates for indirect costs based on two-part model with gamma distributed Generalized Linear Model as second part

Cost for inpatient treatment are included in the adjustment variables baseline direct costs and baseline total costs but did per definition not incur during the study period, hence adjusted costs deviate substantially from unadjusted costs

*AMD* Adjusted mean difference, *CI* Confidence interval, *M* Mean, *MADRS* Montgomery-Asberg Depression Rating Scale, PHQ-9 = Patient Health Questionnaire,* SD* Standard deviation

#### Sensitivity analyses

Within SA_1_ mean unadjusted t0 scores of MADRS and PHQ-9 did not differ from the values observed in the main analysis. At t1 AMDs for MADRS (− 2.71; [95%-CI -5.82; 0.57]) and PHQ-9 (− 1.45; [95%-CI: − 5.93; 0.58]) were slightly more favorable than in the main analysis without being significant. SA_2_ almost perfectly mirrored the results of the main analysis.

### Observable data on health care utilization

Unadjusted, unimputed data on health care service utilization and frequency mirror high relevance of psychiatrists’ care and individual psychotherapy in BPT and CBT, during the 13 weeks prior to study entry (t0) and during the on average 11.5 weeks of intervention (t1) [see Table 3]. Utilization quotas and frequencies for all sub-categories were comparable between both groups.

**Table 3:** Observed raw data on and utilization frequency at baseline (t0) and end of the intervention (t1) in both study groups

	T0 – period 13 weeks prior to intervention						T1 –intervention period (Ø 11.5 weeks)					
	User Quota			Ø Utilization Frequency			User Quota			Ø Utilization Frequency		
	BPT (***n*** = 79)	CBT (***n*** = 77)	***p***-value	BPT (***n*** = 79)	CBT (***n*** = 77)	***p***-value	BPT (***n*** = 72)	CBT (***n*** = 68)	***p***-value	BPT (***n*** = 72)	CBT (***n*** = 68)	***p***-value
General practioner (vist)	36.7%	37.7%	0.90	0.8	1.0	0.63	22.2%	23.5%	0.85	0.4	0.7	0.68
Psychiatrist (visit)	62.0%	57.1%	0.53	1.6	1.8	0.77	54.2%	39.7%	0.09	1.1	0.9	0.11
Individual psychotherapy (session)	53.2%	49.4%	0.63	6.7	6.2	0.85	48.6%	42.6%	0.48	6.2	5.4	0.87
Group psychotherapy (session)	5.1%	1.3%	0.18	0.6	0.2	0.18	4.2%	0.0%	0.09	0.5	0	0.09
Anti-depressant (prescriptions)	41.8%	40.3%	0.85	−/−	−/−	−/−	11.1%	13.2%	0.7	−/−	−/−	−/−
Rehabilitation (days)	−/−	−/−	−/−	−/−	−/−	−/−	−/−	−/−	0	−/−	−/−	−/−
Psychiatric day clinic (days)	−/−	−/−	−/−	−/−	−/−	−/−	−/−	−/−	0	−/−	−/−	−/−
Psychiatric hospital (days)	7.6%	15.6%	0.12	2.4	6.7	0.11	−/−	−/−	0	−/−	−/−	−/−
Working days lost	44.3%	37.7%	0.29	14.8	12.8	0.36	31.9%	33.8%	0.86	6.8	6.6	0.95
Reduced working hours	10.1%	10.4%	0.93	12.3	12.3	0.91	20.8%	17.6%	0.49	43.3	35.1	0.53
Early retirement (days)	13.9%	11.7%	0.61	9.4	7.6	0.61	13.9%	13.2%	0.88	8.3	8.0	0.89

Sample sizes refer to participants with valid answers at the distinct time points. Within BPT t1 data of 7 individuals were completely missing, within CBT t1 data of 9 individuals were completely missing. As further single missing items did not occur, valid answers are the same for each sub-category

### Costs

**Fig. 1:**
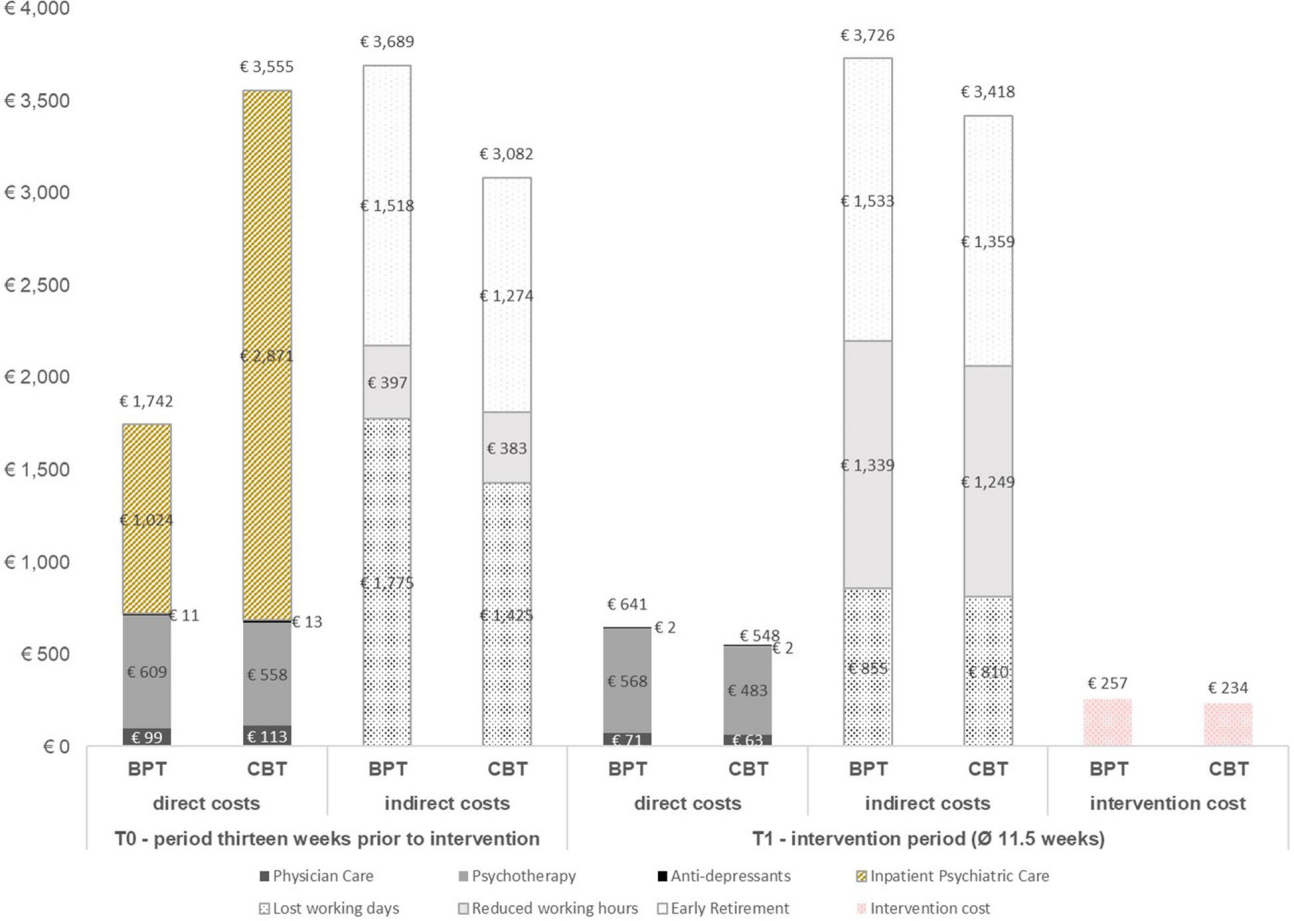
Unadjusted costs at baseline (t0) and end of the intervention (t1)

At t0 unadjusted total costs amounted to €5432 (SD = 6414) in BPT [see Fig. 1], thereof €3689 (SD = 4284) indirect costs. In CBT, corresponding values were similar, with total costs of €6637 (SD = 10,512), thereof €3082 (SD = 4241) indirect costs. There was a huge but not significant difference regarding costs for inpatient psychiatric care (BPT = €1023 (SD = 4645) | CBT = €2871 (SD = 7895)). Until t1, BPT incurred intervention costs of €257 (SD = 95) and CBT incurred intervention costs of €234 (SD = 86). Adjusted total t1 costs were €6019 [95%-CI 4255; 8620] in BPT and €5266 [95%-CI 3684; 7972] in CBT. The resulting AMD of €754 [95%-CI: − 1279; 2737] was not significant (See Table 2).

#### Sensitivity analyses

SA_1_ yielded lower unadjusted t0 costs for BPT and higher unadjusted t0 costs for CBT. Intervention costs increased by approximately €35 in both groups. Adjusted total t1 costs declined by 15% for BPT and by 14% for CBT. In consequence, compared with the main analysis the AMD reduced (€591; [95%-CI €1596; 2878]) without becoming significant. SA_2_ did not substantially affect unadjusted t0 costs. Adjusted t1 costs declined by 8% for BPT but remained stable for CBT. The AMD amounted to about one third of the value observed in the main analysis (€279; [95%-CI -1777; 2332]) and was not significant.

### Cost-effectiveness

**Fig. 2:**
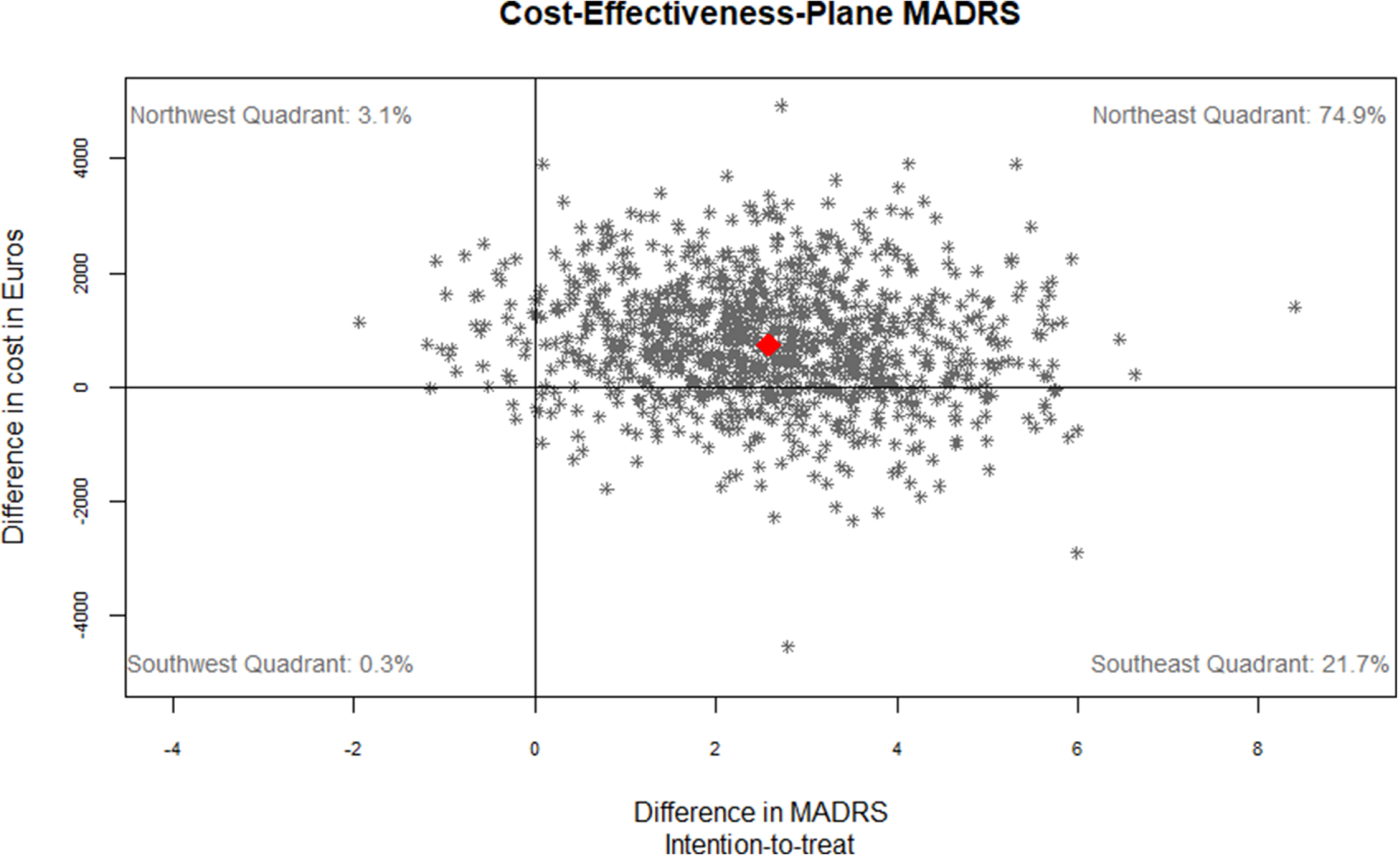
Cost-Effectiveness Plane for 1 MADRS point gained in the ITT sample based on adjusted parameters. Adjusted for baseline values, depression severity, sex, study site, and wave

**Fig. 3:**
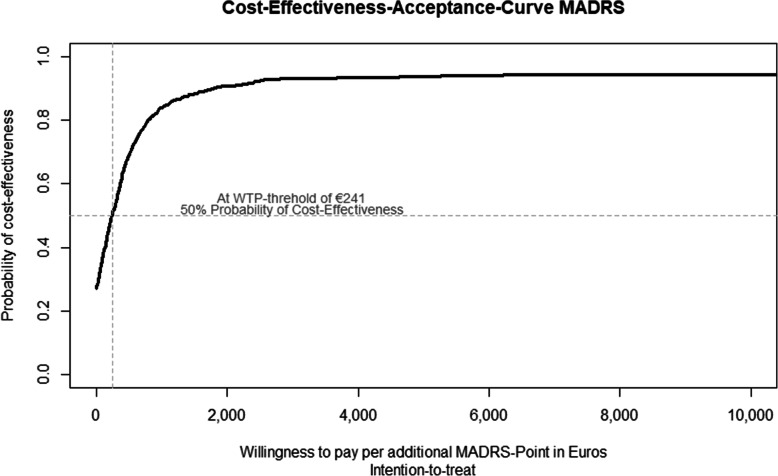
Cost-Effectiveness-Acceptance-Curve for 1 MADRS point gained in the ITT sample based on adjusted parameters. Based on incremental costs and effects adjusted for baseline values, depression severity, sex, study site, and wave

>At t1, the ICER was €288 [95%-CI: − 1800; 3409] per MADRS-point, with 74.9% of bootstrap replications located in the north-east quadrant of the cost-effectiveness plane, which indicates higher effects at higher cost. Another 21.7% of replications fell into the south-east quadrant that indicates dominance of BPT (see Fig. 2). At a WTP-threshold of €241 per unit of MADRS change BPT had a 50% probability of being cost-effective (see Fig. 3).

**Fig. 4:**
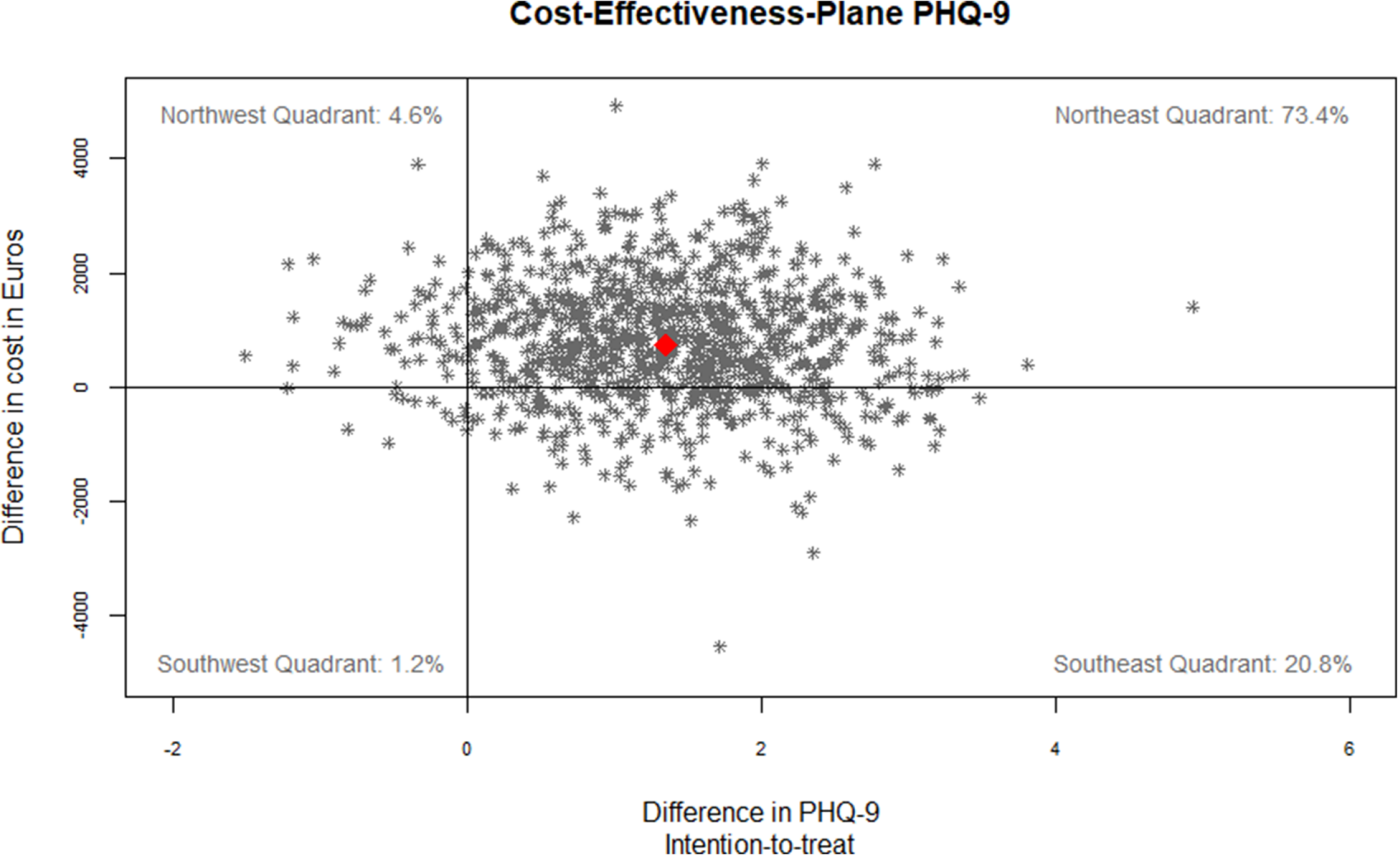
Cost-Effectiveness Plane for 1 PHQ-9 point gained in the ITT sample based on adjusted parameters. Adjusted for baseline values, depression severity, sex, study site and wave)

**Fig. 5:**
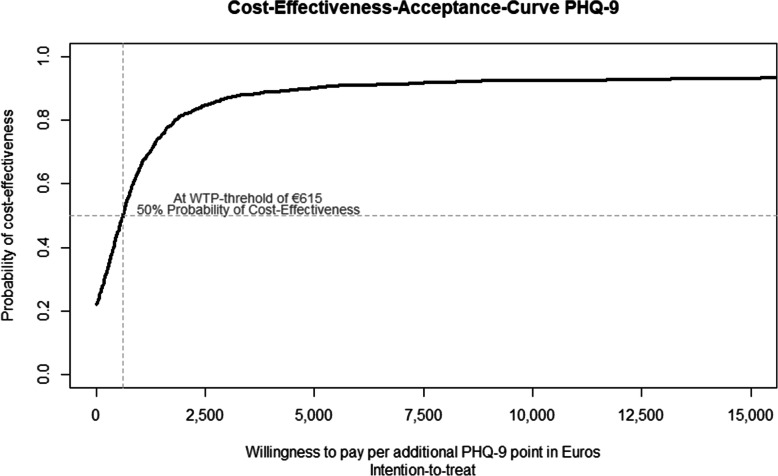
Cost-Effectiveness-Acceptance-Curve for 1 PHQ-9 point gained in the ITT sample based on adjusted parameters. Based on incremental costs and effects adjusted for baseline values, depression severity, sex, study site, and wave

Regarding PHQ-9, the ICER at t1 was €550 [95%-CI: − 3136; 8768] per additional point, with 73.4% of bootstrap replications falling into the north-east and 20.8% in the south-east quadrant of the cost-effectiveness plane (see Fig. 4). At a WTP-threshold of €615 per unit of PHQ-9 change BPT had a 50% probability of being cost-effective (see Fig. 5).

#### Sensitivity analyses

SA_1_ confirmed the results of the main analysis: The ICER for both, MADRS (€218, [95%-CI -2667, 2524]), and PHQ-9 (€412, [95%-CI: − 6423; 6087]) was slightly reduced. Furthermore, each cost-effectiveness plane presented a slightly more favorable distribution of bootstrap replications (MADRS: north-east: 69.0%, south-east: 25.8% | PHQ-9: north-east: 66.8%, south-east: 24.8%) [see Figs. S2 & S4]. A 50% probability of BPT being cost-effective was reached again at €241 for MADRS [see Fig. S3] and at €460 already PHQ-9 [see Fig. S5].

In SA_2_ the ICER for both, MADRS (€109; [95%-CI: − 1773; 2700]) and PHQ-9 (€200, [95%-CI: − 2826; 5214]) was only about one-third of the value obtained in the main analysis. The cost-effectiveness planes demonstrated a considerably higher share of replications in the south-east quadrant (MADRS: 36.0%, PHQ-9: 35.1%) [see Figs. S6 & S8]. A 50% probability of cost-effectiveness was reached at the much lower WTP thresholds of €136 for MADRS and of €234 for PHQ-9 [see Figs. S7 & S9].

## Discussion

This paper investigated cost-effectiveness of BPT using group-based CBT as comparator. At end of the intervention period, symptoms of depression severity had developed more favorably in BPT (MADRS: AMD = -2.58; PHQ-9: AMD = − 1.35).

Looking at the effect side, within both groups t0 values indicated on average moderate and t1 values on average mild depression. We observed within group effect sizes (Cohen’s d) of 0.89 for MADRS and of 0.85 for PHQ-9 in BPT, which are considered large effects. For CBT corresponding values were 0.66 and 0.65, both considered as medium effects [47]. This underlines the effectiveness of both interventions but also emphasizes the slight preferability of BPT. Indeed, the observed AMDs for MADRS and PHQ-9 were close to clinical relevance (MIDs: MADRS: − 3.0 points; PHQ-9: − 1.9 points).

Additional costs amounted to €754 and were only to minor extent driven by intervention costs per se (unadjusted mean difference: €23). This finding has to be interpreted against the following caveat: t0 costs for inpatient psychiatric care in CBT were almost three times as high as in BPT, and corresponding costs at t1 were set 0 per definition (admission to psychiatric wards as a reason for exclusion from the study). This creates a substantial regression to the mean effect on disadvantage of BPT because of adjustment for baseline costs.

Looking at the distinct cost components at t1, indirect costs were by trend lower in BPT. Furthermore, structures of indirect costs changed because of a shift from depression-related sick-leaves to part-time work-related productivity loss. Per se, part-time work is other than sick leave predictable and enables the employers to take measures to compensate for the resulting productivity loss (e.g. employing additional staff, raise weekly hours of existing staff). In consequence, after a certain friction time factual productivity loss (i.e., indirect costs) in context of reduced working time might be mitigated. Direct costs were slightly increased in BPT as spending for psychiatrists’ care and concomitant psychotherapy was lower in CBT.

Cost-effectiveness analyses yielded ICERs of €288 per one point improvement of MADRS and of €550 per one point improvement of PHQ-9. These estimates ought to be discussed against a broader context, as reference studies are lacking given the innovative nature of BPT. Regarding our comparator CBT a meta-analysis already documented that cost-utility analyses consistently claim its cost-effectiveness when contrasted with community-referral, usual care, and medication alone [48]. Regarding the comparative effectiveness of individual vs. group-based CBT evidence is inconclusive. An early piece of research argued that in depression treatment group-based CBT might be the more cost-effective format, but the methodological quality of the underlying studies was quite heterogeneous [49]. Contrasting BPT against the cost-effective comparator CBT instead of the RCT’s active control EP is in line with the concept of the efficiency frontier suggested by the German National Institute for Quality and Efficiency in the Health Care Sector [50]. It is however per se more challenging than demonstrating cost-effectiveness against a “placebo-therapy”. As CBT is only one of several recommended therapies for depression [2], the choice of the comparator might have substantial impact on cost-effectiveness estimates.

Furthermore, there is no established WTP-threshold for non-QALY cost-effectiveness estimates and therefore societal WTP for a change in symptoms of depression severity – mirrored by MADRS and PHQ-9 – is unknown: An early US-based study suggests that individuals with depression are willing to invest around 9% (i.e. $270 at 1996–1998 values) of their monthly net household income for participating in a six-months disease management program that achieves freedom of symptoms [51]. Another US-based study concluded that individuals with major depressive disorder were willing to invest about 15% (i.e. $676) of their net income over a one year timeframe to receive treatment with an anti-depressant that cures without side effects [52]. Based on a German net household income of €3399 per month in 2017, this creates a range of €1835 to €6118 as presumable WTP for remission. Within our study population achieving remission would on average have required an improvement of 14 MADRS points and of 6 PHQ-9 points. Hence, if the upper threshold holds, there is good reason to assume that BPT cost-effectively achieves a one unit change of MADRS and PHQ-9. However, if the lower threshold holds, this is only the case for SA_2_. In Germany, every person diagnosed with a mental illness can seek psychotherapeutic treatment without copayment within the scope of the SHI. In this context expenses for e.g. short-term individual CBT amount to about €2500 [41]. Given this framework, we assume a comparatively high societal WTP for improvement of symptoms of depression as well.

The results are robust in our SAs with slight improvements regarding PHQ-9. The PP analysis (SA_1_) suggests that efforts to foster adherence to BPT are promising to improve cost-effectiveness. The outlier-corrected analysis (SA_2_) points to the subpopulation without previous extensive depression treatment as potential key target group for BPT. We conclude this from the fact that both excluded individuals had a history of ongoing psychiatrists’ and psychotherapists’ treatment at baseline that continued throughout the study and furthermore reported previous depression-related inpatient stays taking place before the baseline assessment period.

The results presented must be interpreted against some caveats: First, our analyses focused on disease-specific instead of all-cause health care utilization. As individuals might have more issues to classify e.g. a physician visit as depression-related than to remember a physician visit at all, there might be some misclassification. Second, focusing on disease-specific utilization and costs disregards synergistic effects. We assume that physical activity has not only a beneficial impact on depressiveness but also on the cardio-vascular and the musculoskeletal system, resulting in reduced costs for treating corresponding complaints, as well. Hence, the cost-effectiveness of BPT is probably underestimated. Third, individuals in both groups were allowed to take part in additional individual psychotherapeutic sessions. Hence the observed effects on MADRS and PHQ-9 cannot be perfectly attributed to either BPT or CBT. As utilization of alternate psychotherapeutic sessions did not differ between both groups, we are convinced that this effect more rather concerns the observed effect sizes per se than the difference between BPT and CBT. Finally, adjustment for center and wave instead of clustering by center and wave disregards the existing intraclass correlation. This is associated with variance inflation and indeed significant between-group differences might fail to cross the set significance threshold [53].

On the other hand, the broad inclusion criteria and the acknowledgement for concomitant psycho- and pharmacotherapy create a study setting that mirrors real-world conditions quite well. Nevertheless, before nationwide roll-out, generation of broader health economic evidence in context of an SHI pilot project (§§ 63, 64b & 65 Book V of the German Social Code) is advisable as there is uncertainty regarding intervention costs in a real-life setting (different entrance fees of bouldering halls, different expenditures for therapist training, potential new cost components [e.g. administrative costs]).

In conclusion BPT has apparently high potential as alternate strategy for depression treatment. It is at least as effective as group-based CBT and incurs moderate excess costs. However, BPT cannot be unambiguously claimed cost-effective as reference WTP thresholds for improving severity of depression symptoms are lacking. Furthermore, the study-related artefact of substantially differing baseline costs for inpatient psychiatric care hampers a straightforward interpretation of AMDs to some extent. The conducted analyses on the “population”-level also mask, which distinct subgroups of patients profit particularly from BPT. To support efficient, targeted resource allocation, additional research ought to identify corresponding key target groups. Subsequently BPT might be recommended initially for those subgroups. In addition, in future studies an integration of BPT elements into classic CBT can also be discussed, e.g., as part of exposure training, as part of positive activity, or as practical problem-solving training.

## Supplementary Information

**Additional file 1.** .

**Additional file 2.** .

**Additional file 3.** .

**Additional file 4.** .

**Additional file 5.** .

**Additional file 6.** .

**Additional file 7.** .

**Additional file 8.** .

**Additional file 9.** .

**Additional file 10.** .

## Data Availability

All the results supporting our conclusions are contained in the manuscript. The datasets that were used and/or analysed in the current study are available from the corresponding author upon reasonable request after the publication of the results.

## Abbreviations

AMD: Adjusted mean difference
BPT: Bouldering-Psychotherapy
CBT: Cognitive behavioural therapy
CEAC: Cost-effectiveness acceptance curves
CI: Confidence interval
EP: Exercise program
GLM: Generalized linear model
GP: General practitioner
ICER: Incremental cost-effectiveness ratio
ITT: Intention to treat
KuS: Klettern und Stimmung, i.e. “Climbing and Mood”
MADRS: Montgomery-Asberg Depression Rating Scale
MID: Minimal important difference
PP: Per protocol
PHQ-9: Patient Health Questionnaire
RCT: Randomized controlled trial
SA: Sensitivity analysis
SD: Standard deviation
SHI: Statutory health insurance
WTP: Willingness to pay
