# Supplementary figures and images for: Is bouldering-psychotherapy a cost-effective way to treat depression when compared to group cognitive behavioral therapy – results from a randomized controlled trial
Source: BMC Health Serv Res. 2021 Oct 26;21:1162. doi: 10.1186/s12913-021-07153-1 (PMC8549311; doi:10.1186/s12913-021-07153-1)

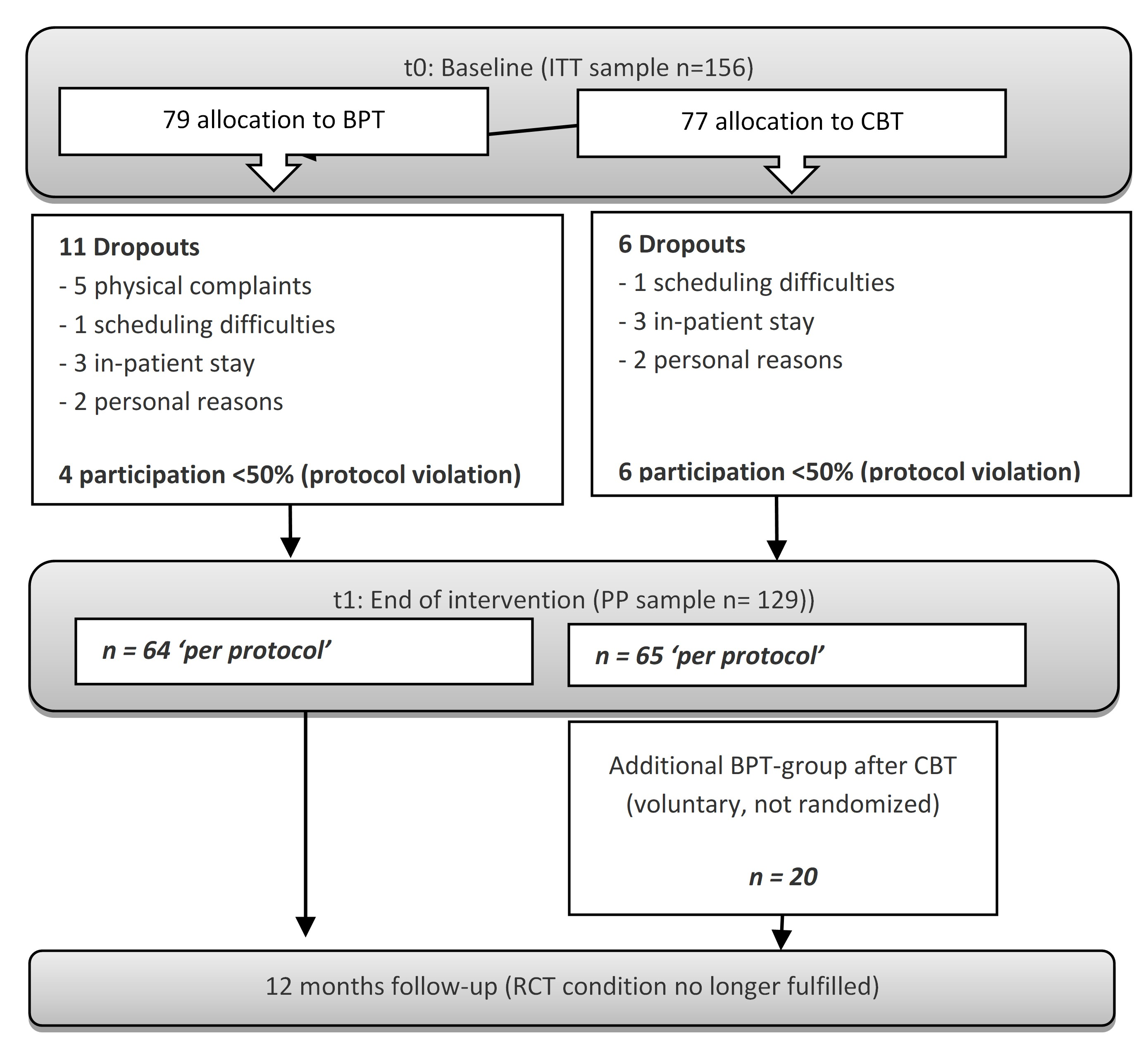

Supplement: Supplementary file 1 — Additional file 1. . [file 12913_2021_7153_MOESM1_ESM.tiff]

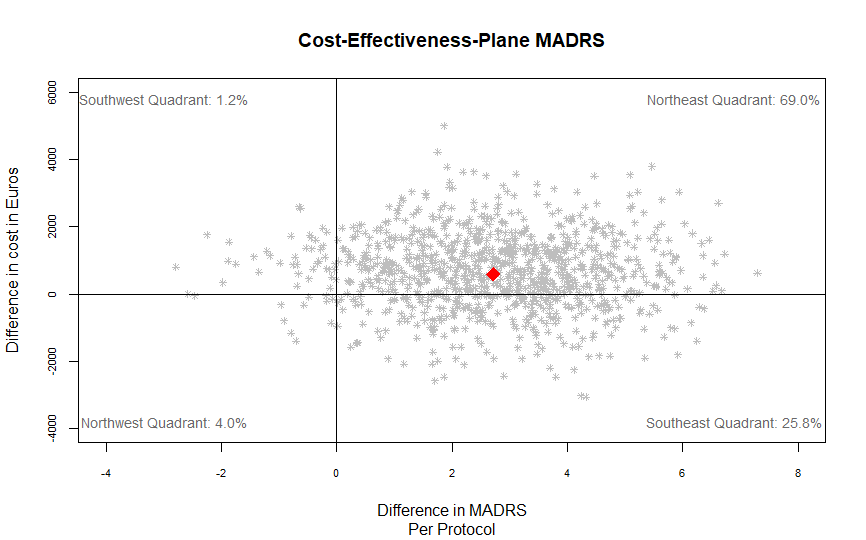

Supplement: Supplementary file 2 — Additional file 2. . [file 12913_2021_7153_MOESM2_ESM.tiff]

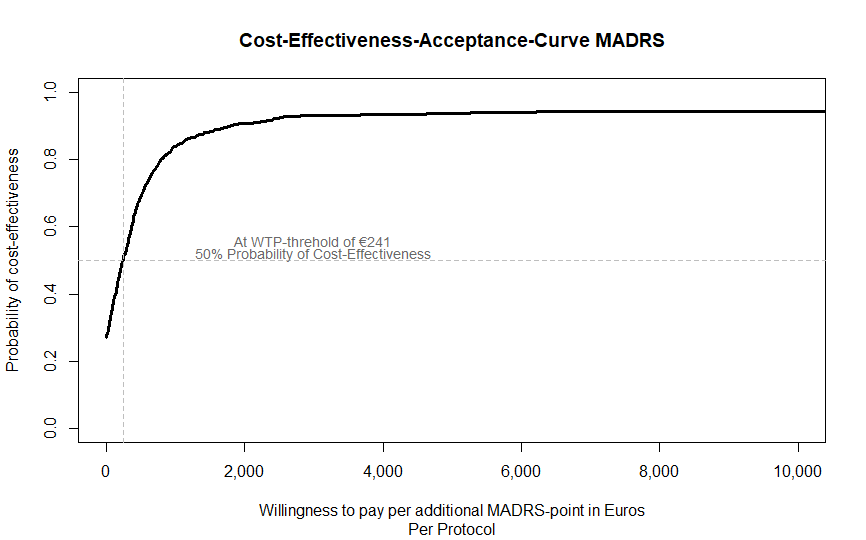

Supplement: Supplementary file 3 — Additional file 3. . [file 12913_2021_7153_MOESM3_ESM.tiff]

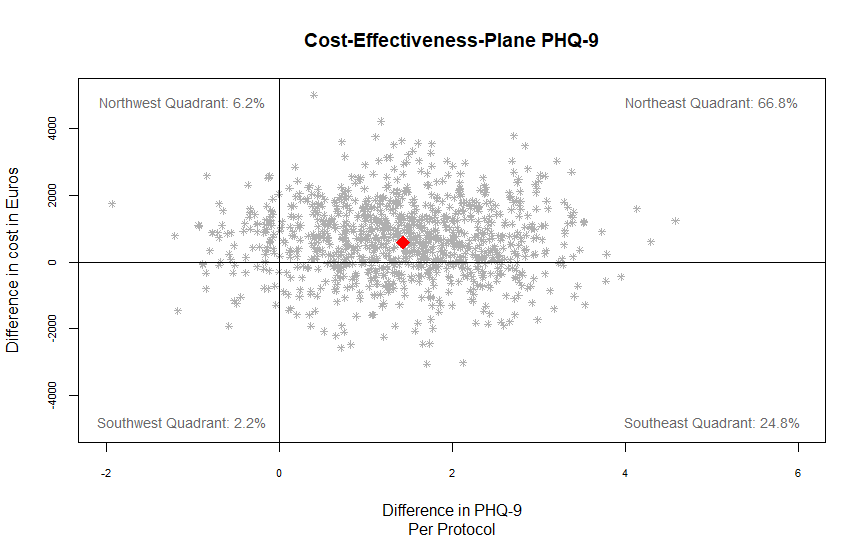

Supplement: Supplementary file 4 — Additional file 4. . [file 12913_2021_7153_MOESM4_ESM.tiff]

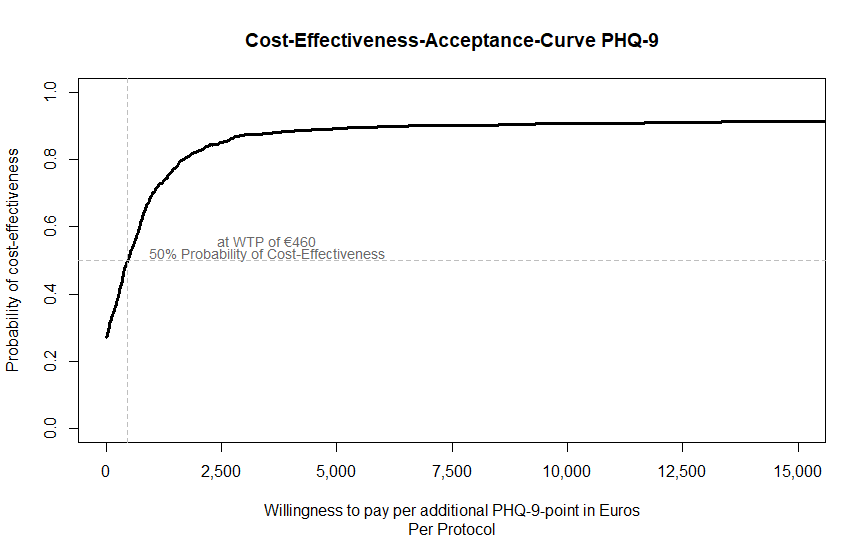

Supplement: Supplementary file 5 — Additional file 5. . [file 12913_2021_7153_MOESM5_ESM.tiff]

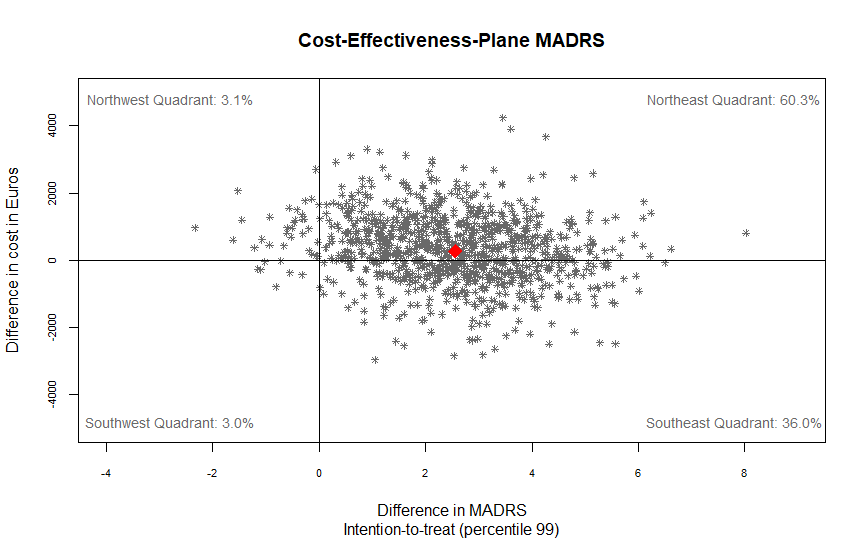

Supplement: Supplementary file 6 — Additional file 6. . [file 12913_2021_7153_MOESM6_ESM.tiff]

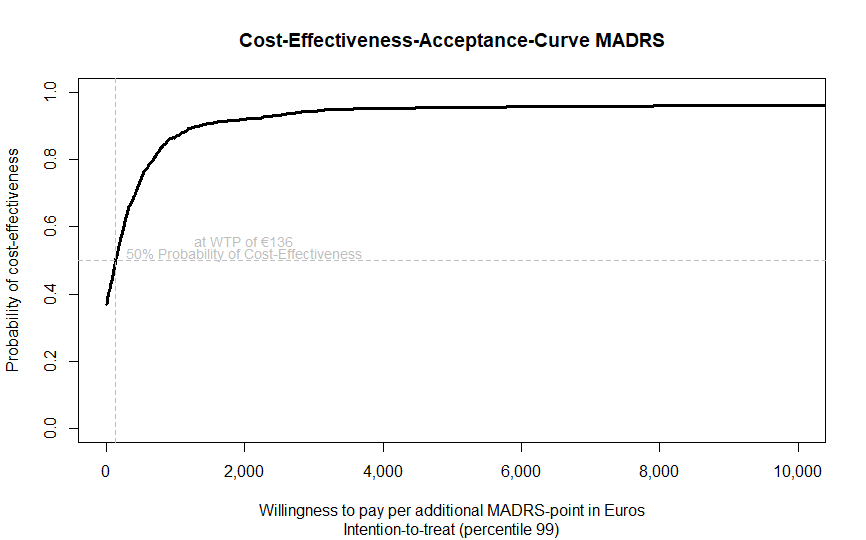

Supplement: Supplementary file 7 — Additional file 7. . [file 12913_2021_7153_MOESM7_ESM.tiff]

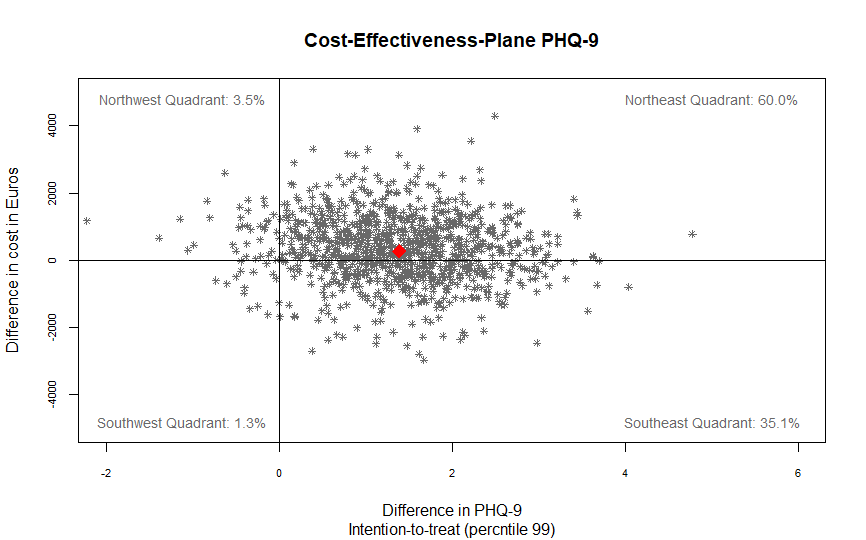

Supplement: Supplementary file 8 — Additional file 8. . [file 12913_2021_7153_MOESM8_ESM.tiff]

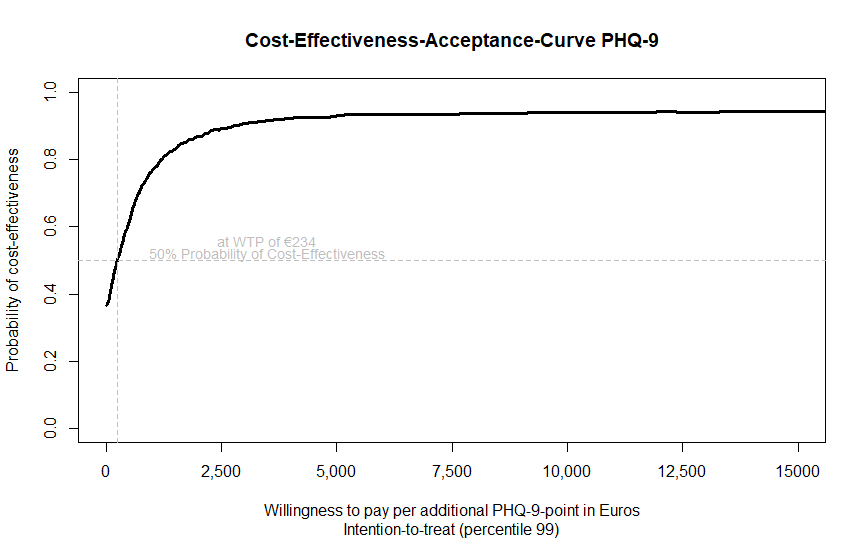

Supplement: Supplementary file 9 — Additional file 9. . [file 12913_2021_7153_MOESM9_ESM.tiff]
