# Supplementary material for: Is bouldering-psychotherapy a cost-effective way to treat depression when compared to group cognitive behavioral therapy – results from a randomized controlled trial
Source: BMC Health Serv Res. 2021 Oct 26;21:1162. doi: 10.1186/s12913-021-07153-1 (PMC8549311; doi:10.1186/s12913-021-07153-1)
Supplement: Supplementary file 10 — Additional file 10. . [file 12913_2021_7153_MOESM10_ESM.docx]

Table S1: German unit costs for health care utilization at base year 2017

| **Domain** | **Value** | **Unit** | **Source** |
| --- | --- | --- | --- |
| General practitioner | €22.13 | Visit | Bock Unit Costs, up-dated |
| Psychiatrist | €50.80 | Visit | Bock Unit Costs, up-dated |
| Individual psychotherapy | €88.56 | Hour | Schedule of fees for psychotherapists |
| Group psychotherapy | €30.64 | Hour | Schedule of fees for psychotherapists |
| Psychiatric hospital | €410.30 | Day | Bock Unit Costs, up-dated |
| Psychiatric day clinic | €266.69 | Day | Bock Unit Costs, up-dated |
| Rehabilitation clinic | €145.63 | Day | Bock Unit Costs, up-dated |
| Lost working hours | €31.10 | Hour | National accounts Employee compensation per hour worked |
| Lost working day | €167.67 | Day | National accounts Employee compensation per employee and potential annual working days |
| Early retirment | €167.67 | Day |  |
|  |  |  |  |
| **Intervention** |  |  |  |
| Group psychotherapy | €30.64 | Hour/patient | Schedule of fees for psychotherapists |
| Entrance bouldering gym | €6.90 | Patient | Average according to study documentation |
| Rental fee climbing shoes | €2 | Patient | Average according to study documentation |
| Safety training therapist | €350 | Trainer | Study documentation |
